# Glycemic Index and Load of Selected Ethiopian Foods: An Experimental Study

**DOI:** 10.1155/2019/8564879

**Published:** 2019-12-24

**Authors:** Nebiyu Dereje, Gadise Bekele, Yemisrach Nigatu, Yoseph Worku, Roger P. Holland

**Affiliations:** ^1^Department of Public Health, Myungsung Medical College, Addis Ababa, Ethiopia; ^2^Department of Medicine, Myungsung Medical College, Addis Ababa, Ethiopia; ^3^Department of Public Health, St. Paul's Hospital Millennium Medical College, Addis Ababa, Ethiopia

## Abstract

**Background:**

Determining the glycemic index and load of foods has significant impact on meal planning for diabetes. However, there is no data on the glycemic index (GI) and glycemic load (GL) of Ethiopian foods. Therefore, the aim of this study was to analyze the glycemic index and glycemic load of Teff Injera, Corn Injera, and White Wheat Bread.

**Methods:**

Experimental study design was conducted among selected healthy adults. Teff Injera, Corn Injera, and White Wheat Bread were selected as test foods for the study, and glucose was used as the reference food. The postprandial glucose concentrations in the blood were recorded at 0, 15, 30, 45, 90, and 120 minutes. The relative glycemic index of each food was calculated, and the presence of statistical difference in glycemic index among the three foods was analyzed.

**Results:**

The mean age of the participants was 23 years (±1.6 years). The glycemic indexes of Teff Injera, White Wheat Bread, and Corn Injera were 36 (low), 46 (low), and 97 (high), respectively, and the glycemic loads were 7 (low), 14 (moderate), and 22 (high), respectively. There was a significant difference in glycemic index and load among the three food items (*p* < 0.001). Teff Injera had a much lower glycemic index and load compared with Corn Injera (*p* < 0.001) and White Wheat Bread (*p* = 0.03).

**Conclusions:**

Teff Injera and White Wheat Bread have low glycemic index and are recommended to be consumed by diabetic patients, whereas Corn Injera has high glycemic index and is not recommended for diabetic patients. Therefore, Teff Injera should be considered globally in the dietary modification programs for diabetes.

## 1. Introduction

The prevalence of diabetes in adult population of Ethiopia was 5.2% in 2018 [1]. Lifestyle modifications including diet are important behavior for diabetes, obesity management, and lipid control, thereby reducing the incidence of metabolic syndrome in patients without diabetes and maintaining the glycemic control in those with both type I and type II diabetes. Carbohydrate intake is a precursor for the rise of glucose in the blood. Therefore, controlling the quantity and quality of carbohydrate used is very critical in the management of diabetes and obesity [2].

Foods that contain carbohydrates are used as a source of energy globally. However, its risk of diabetes and the optimal amount is still controversial. Moreover, since the foods that contain carbohydrate differs in their rate of absorption and rising blood glucose level, attention has been directed to the quality of carbohydrates [3, 4].

Even with the same amount of carbohydrates within the food, the postprandial effect of rising blood glucose varies from one food to another. These variations directed to the growth of the glycemic index and glycemic load concepts. Glycemic index (GI) is an *in vivo* measure of the relative impact of carbohydrate-containing foods on postprandial blood glucose [4], whereas glycemic load (GL) is defined as the product of the GI value of a food and its carbohydrate content; GL incorporates both the quality and quantity of carbohydrate consumed [5–9].

According to the World Health Organization (WHO) recommendations for the prevention and control of diabetes, modifications in diet and an exercise of 30 min/day in a person with impaired glucose tolerance can have an impact of preventing or delaying the development of diabetes by 58% [2, 3]. Despite of the fact that dietary modifications and meal planning does have an impact on the control of diabetes mellitus, the glycemic index of common Ethiopian foods has not been determined. Therefore, we selected staple foods of Ethiopia, namely, Injera (Amharic for bread), both the Teff and Corn varieties, and Wheat Bread to determine their glycemic index and load. The findings of this study can be used by diabetic and obese patients, along with physicians and dieticians for meal planning and management of diabetes mellitus and obesity.

## 2. Materials and Methods

### 2.1. Study Setting, Design, and Population

An experimental study among clinically healthy nondiabetic medical students from Myungsung Medical College was conducted from December 2016 to January 2017. The College has well-organized laboratories which can also be utilized for research activities. At the time of study, there were more than one hundred medical students in the College. Ten volunteer healthy nondiabetic adult students were recruited into the study based on the Food and Agriculture Organization (FAO) and the World Health Organization (WHO) 1998 glycemic index testing protocol [8–10].

### 2.2. Data Collection Tools and Procedures

Ethical approval of this study was obtained from the Institutional Review Board of Myungsung Medical College and Addis Ababa Health Bureau. Informed consent was obtained from the participants to ensure their voluntary participation in the study. The participants' demographic data, vital signs, and body mass index (BMI) were obtained. The demographic data was obtained through questionnaire, and the vital signs were measured by the trained personnel using physical examination, sphygmomanometer, and digital thermometer.

A standard procedure/protocol for GI testing was published by the FAO and the WHO [10]. The current recommendations for the calculations of glycemic index and load recommend that the glycemic response of the reference food used in the study should be repeated three times, so that the average glycemic response value should be taken as a reference [9, 10]. To reduce variations among the subjects and enhance the GI measurement, capillary blood sampling was used [11–14].

The selected test foods were Teff Injera, Corn Injera, and White Wheat Bread. Flour of the test foods was purchased from local supermarket and was baked at home according to the Ethiopian standard agency specification [15, 16]. The reference food for this study was glucose. Administration of 50 g of carbohydrate containing glucose was repeated in three separate occasions in each subject. The test food trial was done once on each study participant on separate occasions. The test foods' proximate composition of ash, moisture, crude fiber, crude fat, and protein were determined. The difference from the result of proximate composition analysis was used to determine the amount of carbohydrate [13]. Then, fifty grams of carbohydrate of each test food was served to the study participants, and they ate within 15 minutes [14]. The meals were served with a glass of water (150 ml) in each of six trials [10].

Blood glucose level was determined by using glucometer (Accu-Chek Aviva Plus Meter) following standard procedures after all subjects fasted overnight. Their blood sample was collected through finger prick using a lancet at 0, 15, 30, 45, 60, 90, and 120 minutes after hands warmed [2, 10]. Each blood sample was placed on a test strip which was inserted into a calibrated glucometer [2, 9, 10]. The blood samples were collected by two trained laboratory personnel. The trial was conducted on six different days.

## 3. Data Management and Analysis

The researchers entered the data in Microsoft Excel and SPSS version 20 for analysis. Summary statistics for mean and standard errors were computed for each of the meal types and participants.

For calculating the glycemic index, variations in the blood glucose concentration from the baseline blood concentration were taken for each postprandial period [10]. The mean of the respective blood glucose response before and after administering the foods was used to draw a blood glucose response curve for the two-hour period. The blood glucose response curve was obtained by plotting a graph (*x*-axis being the time interval and *y*-axis being the blood glucose concentration at the respective times). The incremental area under the glucose curve (IAUC) was calculated by the trapezoidal method (the sum of the areas of the trapezoids between the blood glucose curve and horizontal baseline going parallel to *x*-axis from the beginning of blood glucose curve at time 0 to the point at time 120 minutes). The incremental area under the glucose curve for the standard glucose was obtained by the mean from the three independent glucose curves. The relative glycemic index of each food was calculated as percent of the mean of individual areas under the glucose response curves, by dividing the IAUC for the tested food by the IAUC for the standard glucose and multiplying by 100 [17, 18]. Then, the glycemic index was categorized as low (0–55%), moderate (56–69%), and high (≥70%) [17, 19]. Moreover, the presence of statistically significant difference in glycemic index among the three foods was analyzed using one-way ANOVA (Scheffe's test) [20]. The difference of GI and GL of Teff Injera from the Corn Injera or White Wheat Bread was analyzed using independent sample *t*-test. The level of statistical significance was set at 0.05 and with corresponding 95% confidence interval.

The glycemic load of individual food items was calculated by multiplying a food's GI by the number of digestible carbohydrates in a given serving [11]. Then, the glycemic load of the food items was categorized as low (0–10%), moderate (11–19%), and high (≥20%) [17, 18].

## 4. Results

Ten healthy volunteer medical students participated in the study (six females and four males). The mean age of the participants was 23 years (SD ± 1.6 years). The proximate analyses of the test foods are listed in Table 1. White Wheat Bread has the highest carbohydrate (31.6) and the lowest fat content (1.3) from the other test foods.

## 5. Glucose Response Area of the Test Foods

**Figure 1:**
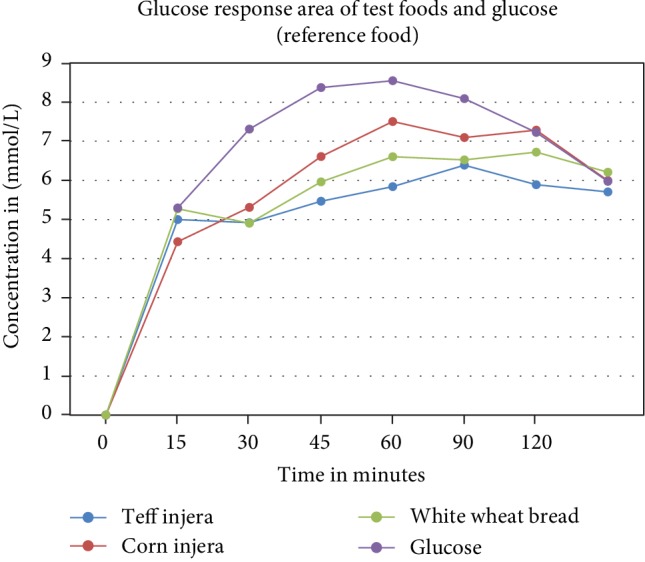
Glucose response curve of the Teff Injera, Corn Injera, and White Wheat Bread and the reference food.

The blood glucose level after administered Teff Injera, Corn Injera, and White Wheat Bread reached a glycemic peak value at 60, 45, and 90 minutes, respectively (Figure 1). There was a significant difference in the blood glucose increment at different time intervals when comparing *Teff Injera*, *Corn Injera*, *and White Wheat Bread with glucose* (*p* < 0.001).

## 6. The Glycemic Index of Teff Injera, Corn Injera, and White Wheat Bread

The mean glycemic index value of Teff Injera was found to be 35.6 (SD ± 17.0, 95% CI 26.2–46.1), which classifies it as a food with low glycemic index. The mean glycemic index value of Corn Injera was found to be 97.4 (SD ± 34.1, 95% CI 79.9–117.9), which classifies it as a food with high glycemic index. The mean glycemic index value of White Wheat Bread was found to be 50.7 (SD ± 23.9, 95% CI 47.9–65.7), which classifies it as a food with low glycemic index (Table 2). There was a significant difference in glycemic index among the three food items (*p* < 0.001). Teff Injera clearly had a much lower glycemic index compared with Corn Injera (*p* < 0.001) and White Wheat Bread (*p* = 0.03).

## 7. Glycemic Load of Teff Injera, Corn Injera, and White Wheat Bread

Glycemic food of the foods was calculated by multiplying a food's GI by the number of digestible carbohydrates in a given serving (Table 3). Accordingly, the glycemic loads of Teff Injera, Corn Injera, and White Wheat Bread were 7.2, 22.3, and 15.9, respectively.

## 8. Discussion

The glycemic index and load of foods are important in the management of hyperglycemia and minimizing need for exogenous insulin in type I diabetes and the presence of hyperinsulinemia in type II [2, 5]. Foods with low glycemic index and load are recommended for diabetic patients, and those foods with high glycemic index and load are not recommended for diabetic patients [4, 13]. Therefore, it is important to know the glycemic index and glycemic load of commonly used foods in a local context, as food processing is one of the main factors which contribute to the variation on the glycemic index and glycemic load of foods [7].

In recent years, the glycemic indexes and loads of foods are used crucial elements in the prevention of diabetes, obesity, dyslipidemia, metabolic syndrome, cardiovascular disease, and some cancers such as colon cancer [5]. Two theories are explaining about how high glycemic index and load foods increase food intake. The first is that it is due to the rise in blood glucose, and the second is that it is due to high insulin response [5, 8, 21].

The principal finding of this study is that Teff Injera and White Wheat Bread had low glycemic index and glycemic load, whereas Corn Injera had high glycemic index and glycemic load. However, a study conducted to determine the glycemic index of selected Ethiopian foods in mice revealed the glycemic index of Teff Injera, Corn Injera, and White Wheat Bread to be 35 (low GI), 43.4 (low GI), and 57 (moderate GI), respectively [22]. The discrepancy in the glycemic index of Corn Injera and White Wheat Bread might be due to the difference in the food processing among the studies. In Ethiopia, there are two main varieties of corn and wheat grown [15, 16]. Different varieties might have different glycemic index and load, but this has not been studied to our knowledge. Moreover, the difference in the findings of the glycemic index between the two studies can be explained by the difference in the study participants used (mice vs. human).

This study showed the GI of White Wheat Bread to be low as compared to the international GI food table of different countries, which ranges from 59 to 89 [23]. The discrepancy could be attributed to the difference in the food processing and high-fiber content of the foods. Teff Injera, which is commonly used food in Ethiopia, has both low GI and GL values. Teff Injera has the lowest carbohydrate, low fat, and relatively higher fiber content than the other two test foods which contribute to its low GI and GL values.

High-carbohydrate-containing diets are suggested for those with diabetes and hyperlipidemia, but the quality of carbohydrate should be also considered for determining the metabolic response to such diets. Carbohydrate-containing foods with high glycemic indexes can result into rise in blood glucose and insulin concentration in the blood, whereas those with low GI foods increase carbohydrate without causing these adverse effects [17].

This study could assist food manufacturers and processors to develop a greater range of low GI and GL processed foods from African farm products. The findings have obvious importance in designing balanced dietary and therapeutic interventions for diabetic patients and others with clinical conditions demanding carbohydrate restriction, such as cardiovascular diseases and obesity [5]. However, it is to be noted that more studies on the GI and GL of locally consumed foods are very important to produce evidence that can guide the use of GI and GL for the prevention and control of diseases.

## 9. Conclusions

Daily diet should be predominated by products that contain slow-digestible carbohydrates. Teff Injera, which is predominantly used in Ethiopia, had a low glycemic index and load. Therefore, Teff Injera is a safe food for diabetic patients, and it can be grown in many drier areas of the world (e.g., Northern United States, Poland, and Western Russia). It should be considered globally in the dietary modification programs for diabetes. Corn Injera had high GI and GL; it is not recommended for diabetic patients.

## Figures and Tables

**Table 1:** Proximate analysis of the test foods.

Test foods	Moisture (%)	Crude fiber (gm)	Protein (gm)	Fat (gm)	Ash (gm)	Carbohydrate (gm)
Teff Injera	62.95	2.58	9.94	1.34	2.99	20.20
Corn Injera	63.24	2.08	8.90	1.39	1.44	22.95
White Wheat Bread	50.34	2.88	12.50	0.75	1.97	31.56

**Table 2:** Glycemic index of Teff Injera, Corn Injera, and White Wheat Bread.

Participants	Glycemic index (%) of Teff Injera	Glycemic index (%) of Corn Injera	Glycemic index (%) of White Wheat Bread
1	53.9	68.5	43.3
2	33.5	74.4	61.2
3	37.5	75.7	25.9
4	25.1	176.7	50.6
5	33.3	127.8	27.2
6	43.8	62.3	26.0
7	30.1	93.5	47.7
8	16.1	99.9	88.5
9	13.2	107.1	92.5
10	70.0	87.7	44.4
	Mean = 35.6 (SD ± 17.0)	Mean = 97.4 (SD ± 34.1)	Mean = 50.7 (SD ± 23.9)

**Table 3:** Glycemic load of test foods.

Test foods	Serving size (gm)	Glycemic index (%)	Glycemic load	GL ranking
Teff Injera	20.2	35.6	7.2	Low
Corn Injera	22.9	97.3	22.3	High
White Wheat Bread	31.5	50.7	15.9	Moderate

## Data Availability

The data used to support the findings of this study are available from the corresponding author upon request.

## Abbreviations

ANOVA: Analysis of variance
CI: Confidence interval
FAO: Food and Agriculture Organization
GI: Glycemic index
GL: Glycemic load
IAUC: Incremental area under the glucose curve
IRB: Institutional review board
MMC: Myungsung Medical College
SPSS: Statistical Packages for Social sciences
WHO: World Health Organization.

## Supplementary Materials

Supplementary Materials The supplementary material is a dataset of the study.
